# On the Chronological Structure of the Solutrean in Southern Iberia

**DOI:** 10.1371/journal.pone.0137308

**Published:** 2015-09-10

**Authors:** João Cascalheira, Nuno Bicho

**Affiliations:** ICArEHB, FCHS, Campus de Gambelas, Universidade do Algarve, 8005–139, Faro, Portugal; University of Oxford, UNITED KINGDOM

## Abstract

The Solutrean techno-complex has gained particular significance over time for representing a clear demographic and techno-typological deviation from the developments occurred during the course of the Upper Paleolithic in Western Europe. Some of Solutrean’s most relevant features are the diversity and techno-typological characteristics of the lithic armatures. These have been recurrently used as pivotal elements in numerous Solutrean-related debates, including the chronological organization of the techno-complex across Iberia and Southwestern France. In Southern Iberia, patterns of presence and/or absence of specific point types in stratified sequences tend to validate the classical ordering of the techno-complex into Lower, Middle and Upper phases, although some evidence, namely radiocarbon determinations, have not always been corroborative. Here we present the first comprehensive analysis of the currently available radiocarbon data for the Solutrean in Southern Iberia. We use a Bayesian statistical approach from 13 stratified sequences to compare the duration, and the start and end moments of each classic Solutrean phase across sites. We conclude that, based on the current data, the traditional organization of the Solutrean cannot be unquestionably confirmed for Southern Iberia, calling into doubt the status of the classically-defined type-fossils as precise temporal markers.

## Introduction

In the framework of the European Upper Paleolithic the Solutrean techno-complex emerges as one of the most unique and intriguing cultural phenomena. Geographically confined to Southwestern France and the Iberian Peninsula, and occurring within a moderately short chronological range (c. 25–19 ka cal BP) that roughly matches the course of the Last Glacial Maximum (LGM), it represents a clear disruption from the previous pan-European techno-complexes.

Rather exceptional, the feature that more noticeably isolates the Solutrean among the Late Pleistocene techno-typological variability is the suite of technological innovations developed for the manufacture of lithic armatures using unifacial and bifacial invasive flat retouch.

Solutrean stone projectiles are the foremost development of this period and a wide variety of these implements are recognized, including several types of foliates, shouldered and tanged morphologies. All of these were most definitely used as tips for thrust and/or projectile weapons, likely as bow and arrow technology [1], even though some may have occasionally been used as knives [2, 3].

Due to its uniqueness, Solutrean weaponry have received a lot of attention over time. Its techno-typological characterization has been the keystone in some of the most debated Solutrean topics, such as the techno-typological origins (i.e. external influence vs. indigenous development–see e.g. [4, 5, 6, 7, 8, 9] *contra* [10, 11, 12, 13]), the possible demographic and cultural expansion towards North America during the LGM (e.g. [14, 15, 16, 17] *contra* [18, 19]), or the internal chronological organization of the techno-complex (e.g. stage subdivisions vs. functional variability in Northern Iberia–see e.g. [20, 21, 22] *contra* [23, 24]).

Regarding this latter topic, the presence/abundance and absence/rarity of certain point types along the stratigraphic sequences of Solutrean key-sites have allowed the application, for most regions, of the classical chronological subdivision of the techno-complex into Lower, Middle and Upper phases, as originally defined by Smith [25] in France.

Across Iberia, the tripartite chronological organization of the techno-complex has, over the years, been recurrently claimed based on data coming from excavation of new sites and on the reassessment of stratigraphic sequences [9, 10, 12, 13, 20, 21, 22, 26, 27, 28].

According to the classical model, each phase is marked by the dominant presence of specific projectiles, truly working as fossil-types. Although radiocarbon data has played an important role in this scenario, frequently defining the limits for the existence *terminus ante quem* or *terminus post quem* of the various type-fossils, this model has been supported mostly by the application of the principle of superposition of projectile types in the so-called key-sequences. Some of the sites were, however, excavated long ago with methodologies that could not accurately define clear stratigraphic changes. Unfortunately, only in a few cases, sequences have been critically analyzed for stratigraphic consistency [29]. This is particularly important if we have in mind that given the nature and pattern of Last Glacial climatic oscillations it is to be expected that long stratigraphic series that span the LGM will most probably be affected by erosion and hiatuses, favoring the formation of palimpsests and other important post-depositional problems [11, 30]. Caldeirão cave, in Portuguese Estremadura, is perhaps the most relevant example in this context since, as mentioned elsewhere [31, 32], several problems are associated with the Solutrean sequence, including the unsecure provenance of all the Upper Solutrean type-fossils [28]. At Parpalló (Valencia), Tiffagom [9] highlights also many problems with the individualization of technological patterns along the stratigraphy of the cave due to the apparent complexity of formation processes affecting the Solutrean sequence, which the excavation by Pericot, using rather thick artificial levels, did not help to control.

Furthermore, the lack of generalized in-depth, intra and inter-site, lithic studies and the resultant apparent absence of techno-typological changes (other than for the "typical" implements), from phase to phase, in the Solutrean tool-kits, bias the knowledge, to some extent, and makes it dependent of the historical precedents of the classical scheme.

Several authors have previously questioned the classical organization and its application in several of the Solutrean core-regions of Iberia [23, 24, 31, 33, 34]. None, however, have specifically focused on Southern Iberia as a whole, and used absolute data, from and linking up the various regions, to assess the traditional model.

In this article we review the chronological data currently available for the Solutrean in Southern Iberia focusing, mostly, on how radiocarbon results challenge and/or corroborate the traditional organization of the techno-complex. Through the results obtained from the application of Bayesian Modeling we then discuss some alternative hypothesis for the explanation of the divergences between the classical scheme and the existing radiocarbon determinations.

## Background

### Geography

Based on the distribution of certain types of projectiles, the Solutrean in Iberia is organized into facies that are likely stylistic in nature [35, 36, 37, 38, 39]. This geographical partition can be observed at distinct levels. On one hand, the territory can be subdivided into two macro-regions [40], the Atlantic or Franco-Cantabrian and the Mediterranean. This division is based on the type of retouch used in the manufacture of shouldered points, predominantly flat, invasive and mostly bifacial retouch in the first case, and abrupt retouch in the second. On the other hand, the existence of specific projectile morphologies in specific regions allows for a more precise identification of territories. The barbed-and-tanged “Parpalló” points, for example, are one of the defining elements of the Mediterranean facies. This facies is coincident with the territory here defined as Southern Iberia (south of the parallel 40°N) with a distribution concentrated in the coastal strip between the Valencia region and the Portuguese Estremadura [1, 8, 41].

**Fig 1:**
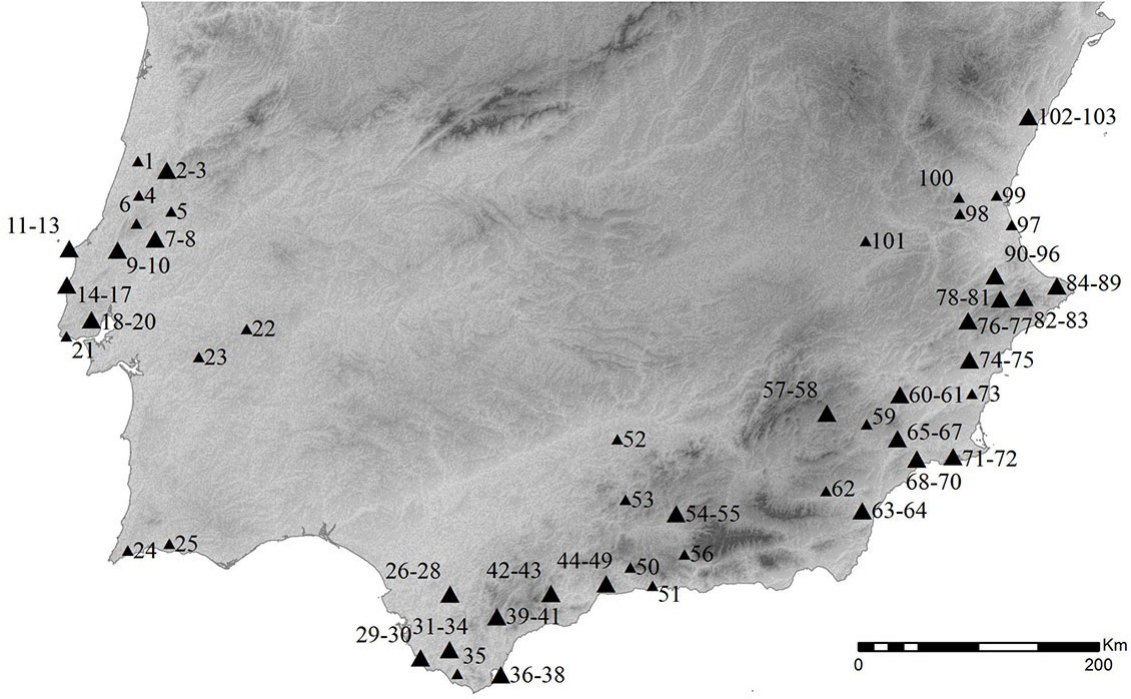
Solutrean sites in Southern Iberia. 1. Ourão; 2–3. Buraca Grande, Buraca Escura; 4. Lagar Velho; 5. Caldeirão; 6. Lapa do Anecrial; 7–8. Almonda, Casal do Cepo; 9–10. Olival da Carneira, Passal; 11–13. Furninha, Casa da Moura, Lapa do Suão; 14–17. Lapa da Rainha, Porto Dinheiro, Baío, Vale Almoinha; 18–20. Gruta de Salemas, Gruta do Correio-Mor, Rua de Campolide; 21. Poço Velho; 22. Monte da Fainha; 23. Gruta do Escoural; 24. Vale Boi; 25. Vala; 26–28. C. Higueral S. Valleja, Llanos de Don Pedro, La Escalera 3; 29–30. La Fontanilla, Casa de Postas; 31–34. Choritto, Cubeta de la Paja, Tajo de la Figuras, C. de Levante; 35. C. del Moro; 36–38. Río Palmones, Sewell's Cave, Gorham's Cave; 39–41. C. del Higueral de Motillas, Abrigo del Bombín, C. de la Pileta; 42–43. Trinidad de Ardales, C. Tajo del Jorox; 44–49. El Bajondillo, Toro, Complejo del Humo, C. del Hoyo de la Mina, C. del Higuerón, C. de la Araña; 50. C. Boquete de Zafarraya; 51.C. de Nerja; 52. Peña Grieta; 53. El Pirulejo; 54–55. C. de Malalmuerzo, Pantano de Cubillas; 56. C. de los Hojos; 57–58. C. de Ambrosio, Chiquita de los Treinta; 59. Barranco de la Hoz; 60–61. Finca Doña Martina, La Boja; 62. Almaceta; 63–64. C. de los Morceguillos, C. del Serrón-Palica; 65–67. Hoyo de Pescadores, C. de Cejo del Pantano, C. de la Moneda; 68–70. C. del Palomarico, Los Tollos, C. Horadada; 71–72. C. Hernández Ros, C. Vermeja; 73. Los Mortolitos; 74–75. Ratla del Bubo, C. del Sol; 76–77. Cantos Visera, Viña de Huesca Tacaña; 78–81. Troncal de la Corona, C. Beneito, C. Negra, Pinaret dels Frares; 82–83. C. de Reinós, C. de Sta. Maria; 84–89. C. del Cendres, C. de les Calaveres, C. Ampla del Cap Gros, C. del Montgó, Abrigo del Capurri, Font de Maria Rosa; 90–96. C. dels Porcs, C. del Llop, C. de les Maravelles, C. del Parpalló, Rates Penaes, C. de Barranc Blanc, C. de les Mallaetes; 97. Volcan del Faro; 98. Covalta; 99. Balsa de la Dehesa; 100. C. del Llentiscle; 101. Palomar; 102–103 Pla de la Pitja, Corral Blanc.

In Southern Iberia the presence of Solutrean materials have been reported in a total of 103 sites (Fig 1). Within this sample, caves and rock-shelters are better represented than open-air locations, as are multi-component sites over single horizon ones.

More than half of the identified sites are, however, surface or mixed contexts and thus suffer from a number of limitations [42], including lack of organic materials, presence of small and highly truncated lithic assemblages and the occurrence of palimpsests of artifacts from different chronologies that, most of the times, are impossible to separate.

Most sites tend to be concentrated in core areas that, historically, were object of more intense survey and excavation. The result is a general impression of geographical hiatuses where, for a long time, the complete absence or the presence of very few sites suggested human passage but not necessarily intensive occupation [43]. During the last two decades those blank areas, such as the case of southern Portugal (or the Côa Valley in northern Portugal), have progressively being punctuated with new sites [44]. This new evidence has contributed to a better understanding of Solutrean settlement and cultural dynamics, clearly emphasizing the importance of peripheral regions [45].

Generically, a total of 4 core areas of Solutrean occupation can be identified within Southern Iberia: Central Portugal, roughly corresponding to the Portuguese Estremadura, with a total of 20 sites; Southern Portugal, where only two sites have been identified with Solutrean levels; Southwestern Spain, roughly coincident with most of Andaluzia and Gibraltar, with a total of 31 sites; and finally, Southeastern Spain, with the largest number of sites (37) encompassing the easternmost territories of Andaluzia, and the provinces of Alicante, Murcia, and Valencia (Fig 1).

### The classical Solutrean sequence model

The first classification of the Solutrean in Southern Iberia was established by Pericot [8] based on the analysis of the materials recovered in the 1930’s excavations at Parpalló cave (Table 1). Pericot proposed a sequence in three major phases (Lower/Protosolutrean, Middle and Upper) and a fourth final stage, called Solutreo-Aurignacian, characterized by a maximum peak in the production of shouldered backed projectiles and by a marked decrease in the presence of the bifacial invasive retouch. Posteriorly, Fullola [46], following this model, readapts the nomenclature of the final stage, assigning it the name by which it is currently known—the Solutreo-Gravettian.

**Table 1:** Classical schemes for the Solutrean in Southern Iberia.

Pericot (1942) [8]	Jordá (1955) [6]	Fortea and Jordá (1976) [47]	Fullola (1979) [38]	Villaverde and Peña (1981) [48], Aura (1986) [49], Rodrigo (1987/88) [50]	Ripoll (1988) [51]	Zilhão (1994) [52]
Lower	Phase I	Initial	Solutreanization	Lower	Lower	Lower
Middle	Phase II	Fully developed	Middle	Middle	Middle	Middle
Upper	Phase III	Evolved I	Upper	Evolved I	Upper	Upper
Solutreo-Aurignacian	Phase IV	Evolved II	Solutreo-Gravettian I	Evolved II	Upper evolved	Solutreo-Gravettian
		Evolved III	Solutreo-Gravettian II	Evolved III		

A few years later, the four stage classification, that had already been confirmed by Jordá’s work [6], would be replaced by a new model based on the materials from Les Mallaetes cave (also located in the Valencian region) [47]. The new template maintained a tripartite organization for the Solutrean, with new names for each phase: Early, Middle and Evolved, while this latter stage was subdivided into three different moments (I, II and III), distinguished by the prevalence, in each one, of different typological elements. Thenceforward, this proposal was corroborated, with very few adjustments, by several other authors working in the Valencia region [9, 48, 49, 50, 53, 54, 55]. Only S. Ripoll [51], followed later by Aura and colleagues [43], proposed a slightly different version of this progression to match the multi-layered Solutrean sequences of Ambrosio and Nerja, respectively.

In Central Portugal, the chrono-stratigraphic model available and generally accepted for the Solutrean was synthesized by Zilhão [37, 52] with the respective updates [11, 28]. The chrono-cultural subdivision is made, once again, under the paradigm of a tripartite division (see also [56]), to which the author added, an initial Proto-Solutrean phase [13, 28].

Overall, according to the classical model the oldest Solutrean phase is the Lower Solutrean, featuring a dominant presence of pointes à face plane with dorsal, invasive, flat retouch and lacking both bifacially-shaped foliate points and tanged types. Contexts attributable to this phase are scarce and constrained to the Mediterranean region (Table 2).

**Table 2:** Phase attribution for Solutrean sites in Southern Iberia (data obtained from [1, 28, 42, 56, 57, 58]). Site numbers match the numbers in Fig 1. Crosses indicate secure presence of diagnostic materials and question marks indicate possible attribution.

	Site	Lower	Middle	Upper	Solutreo-Gravettian
1	Ourão			X	
2	Buraca Grande			X	?
4	Lagar Velho		X		
5	Caldeirão		X	X	
7	Almonda			X	
8	Casal do Cepo		X		
9	Olival da Carneira			X	
10	Passal			X	
11	Furninha			X	
13	Suão			X	
16	Baío			X	
17	Vale Almoinha		X		
18	Salemas			X	
19	Correio-Mor			X	
21	Poço Velho			X	
22	Casa da Moura			X	
22	Monte da Fainha		X		
24	Vale Boi			X	
25	Vala				X
26	Sierra Valleja			X	X
27	Llanos Pedro			X	
29	La Fontanilla			X	?
30	Casa de Postas			?	?
31	El Chorrito			X	X
32	Cubeta de la Paja			X	X
33	Tajo de las Figuras			X	X
34	Cuevas de Levante			X	X
36	Sewell o Cueva “S”			X	X
37	Gorham’s			X	
39	Cueva del Higueral			X	
41	Abrigo del Bombín			?	?
43	Tajo de Jorox			X	
44	Bajondillo		X		X
46	Complejo Humo			X	X
48	Higuerón-Suizo			X	X
50	Zafarraya			X	X
51	Nerja	X	X		X
52	Peña de la Grieta			X	?
53	El Pirulejo			X	X
55	Pantano Cubillas				X
56	Cueva de los Ojos			X	
57	Ambrosio		X	X	X
59	Barranco de la Hoz			X	X
60	Finca Doña Martina			X	
61	La Boja	X		X	X
63	Morceguillos			?	?
64	Serrón la Palica			X	X
65	Hoyo de Pescadores			X	X
66	Cejo del Pantano				X
68	Palomarico			?	?
69	Los Tollos	?			
71	Hernández Ros			X	X
73	Los Mortolitos		?	X	
74	Ratla del Bubo				X
75	Cueva del Sol			?	?
76	Cantos de la Visera			?	
77	Huesa Tacaña		X		
79	Cova Beneito	X	X	X	X
80	Cova Negra			?	?
81	Pinaret dels Frares		X		
85	Les Caraveles			?	?
86	C. Ampla			?	?
87	Cova del Montgó				X
88	Abrigo Capurri			X	X
89	Font de Mª Rosa			X	X
90	C. dels Porcs		X	X	X
91	Cova del Llop			X	X
92	Les Maravelles		X	X	X
93	Parpalló	X	X	X	X
94	Rates Penaes		?	?	
95	Barranc Blanc	X	X	X	X
96	Mallaetes	X	X	X	X
98	Covalta			?	?
99	Balsa de la Dehesa			?	X
100	Cova de Llentiscle			?	?
102	Pla de la Pitja			X	?
103	Corral Blanc			X	?

During the Middle Solutrean, lithic tool-kits are marked by the predominance of laurel-leaves over pointes à face plan, although the latter seem to maintain a relatively important presence within some of the assemblages. A total of 17 contexts have been attributed to the Middle Solutrean (Table 2) and, with exception of Southern Portugal, sites are spread across Southern Iberia.

The number of Upper Solutrean contexts is the highest among all three phases (Table 2). From a typological point of view, Upper Solutrean assemblages are marked by the presence of pointes à face plan, although in much more restricted frequencies, while laurel-leaves maintain almost the same importance as in the previous phase. Tanged and winged “Parpalló-type” points are the most significant implements during this stage, reaching very relevant numbers in many sites, particularly at Parpalló, Mallaetes and Ambrosio [1]. The other type of projectile representative of this phase is the shouldered point with abrupt retouch. Its appearance at this time, excluding the ones ascribed to the Middle Solutrean at Parpalló [1], seems to witness a return to the small abruptly retouched projectiles from the Gravettian. This type of projectile is thought to disappear, in fact, from the archaeological record for more than 3000 years, to become dominant in the final stages of the LGM. It is currently argued that their spreading was from an original focus on the center of the final Gravettian of Eastern Europe [9].

Variation in the presence of stemmed and shouldered projectiles have been the base for a further chronological subdivision of the Upper Solutrean phase. In this perspective the progressive disappearance of the Parpalló-type points and subsequent dominant presence of the small backed shouldered points allowed the definition of an Evolved Upper Solutrean or Solutreo-Gravettian. This phase has, however, fundamental implications to the transition from Solutrean to the Magdalenian, more so than in the internal organization of the Solutrean techno-complex itself. Nonetheless, while in Central and Southern Portugal the Solutreo-Gravettian phase is yet to be proved to exist [59], in SW and SE Spain the top levels from Solutrean sequences such as Nerja, Cendres, Parpalló, Beneito or La Boja have been systematically attributed to the Solutreo-Gravettian [1, 33, 57].

Finally, it is important to mention that, according to the classical organization, since the point types do not replace one another but are instead added on to the existing types, the occurrence of a pointe à face plan does not automatically indicate a Lower Solutrean, whereas the presence of a shouldered point, by definition, will immediately recognize an Upper Solutrean occupation [25, 35].

## Materials and Methods

### Radiocarbon database

A total of 59 radiocarbon determinations are currently available for the Solutrean in Southern Iberia (Table 3). The results come from a total of 18 sites [28, 33, 45, 47, 57, 58, 60, 61, 62, 63, 64, 65, 66, 67, 68, 69, 70, 71, 72], mostly from caves and rock shelters and only two dates come from an open-air occupation in Central Portugal (Vale Almoinha). In the Atlantic facade, Vale Boi, in Southwestern Portugal, is the site with the highest number of radiocarbon determinations, followed by the cave of Caldeirão, in the Portuguese Estremadura.

**Table 3:** Radiocarbon dates for the Solutrean in Southern Iberia.

Site	Level	Lab. Ref.	Age	Deviation	Sample	Method	Phase	Reference
Ambrosio	IV	Gif-9884	21520	120	charcoal	standard	Upper	Jordá et al., 2012 [60]
Ambrosio	IICapa4	Gif-A-II.4	19110	90	charcoal	AMS	Solutreo-Gravettian	Jordá et al., 2012 [60]
Ambrosio	IICapa2	Gif-A-II.2	19170	190	charcoal	AMS	Solutreo-Gravettian	Jordá et al., 2012 [60]
Ambrosio	IIGenerico	Gif-9883	19250	70	charcoal	standard	Solutreo-Gravettian	Jordá et al., 2012 [60]
Ambrosio	II.6	Gif-A-II.6	19300	190	charcoal	AMS	Solutreo-Gravettian	Jordá et al., 2012 [60]
Ambrosio	IICapa1	Gif-A-95577	19950	210	charcoal	AMS	Solutreo-Gravettian	Jordá et al., 2012 [60]
Ambrosio	IICapa1	Gif-A-95576?	20150	200	charcoal	AMS	Solutreo-Gravettian	Jordá et al., 2012 [60]
Bajondillo	9a	AA-34710	19990	480	bone	AMS	Middle	Cortés, 2007 [61]
Beneito	II (ext)	Ua-32243	16180	140	bone	AMS	Middle	Domenech et al., 2012 [62]
Beneito	B2	Ly-3596	16560	480	bone	standard	Upper	Iturbe and Cortell, 1987 [63]
Beneito	IV (ext)	Ua-32244	18275	175	charcoal	AMS	?	Domenech et al., 2012 [62]
Buraca Grande	9	Gif-9502	17850	200	charcoal	AMS	Upper	Aubry et al., 2001 [64]
Caldeirao	H	OxA-1939	19900	260	capra	AMS	Middle	Zilhão, 1997 [28]
Caldeirao	Fatopo	ICEN-295	21200	2300	charcoal	standard	Upper	Zilhão, 1997 [28]
Caldeirao	Fatopo	OxA-1938	20400	270	bone	AMS	Upper	Zilhão, 1997 [28]
Caldeirao	Fc	OxA-2510	18840	200	bone	AMS	Upper	Zilhão, 1997 [28]
Caldeirao	H	OxA-2511	20530	270	bone	AMS	Middle	Zilhão, 1997 [28]
Cendres	XIIB	Beta118024	17230	130	-	AMS	Solutreo-Gravettian	Villaverde et al., 1999 [65]
Cendres	XIIIB	Beta118026	18920	180	charcoal	AMS	Upper	Villaverde et al., 1999 [65]
Cendres	XIIIB	Beta118027	18750	130	charcoal	AMS	Upper	Villaverde et al., 1999 [65]
Finca Dona Martina	4/5	VERA-5101bHS	19180	90	charcoal	AMS	Upper	Zilhão et al., 2011 [58]
Gorham's	III	Beta-184042	18440	160	charcoal	AMS	Upper	Finlayson et al., 2006 [66]
Gorham's	III	Beta-181893	16420	120	charcoal	AMS	Upper	Finlayson et al., 2006 [66]
La Boja	SW18B1	Vera-5788	16580	70	charcoal	AMS	Solutreo-Gravettian	Lucena et al., 2012 [57]
La Boja	SW18B2	Vera-5364-a	16990	70	charcoal	AMS	Solutreo-Gravettian	Lucena et al., 2012 [57]
La Boja	SW18B2	Vera-5364-b	17430	70	charcoal	AMS	Solutreo-Gravettian	Lucena et al., 2012 [57]
La Boja	SW18C	Vera-5365	19390	100	charcoal	AMS	Upper	Lucena et al., 2012 [57]
La Boja	SW18E	Vera-5366	20980	120	charcoal	AMS	Lower	Lucena et al., 2012 [57]
La Boja	SW18E	VERA-5213	20980	110	charcoal	AMS	Lower	Lucena et al., 2012 [57]
Lagar Velho	9	OxA-8419	20200	180	charcoal	AMS	Middle	Zilhão and Trinkaus, 2002 [67]
Mallaetes	III	KnI-918	16300	1500	-	standard	Upper	Fortea and Jordá, 1976 [47]
Mallaetes	II	KN-1/915	19370	105	-	standard	Solutreo-Gravettian	Fortea and Jordá, 1976 [47]
Mallaetes	VI	KnI-920	21710	650	charcoal	standard	Lower	Fortea and Jordá, 1976 [47]
Mallaetes	Va	KnI-919	20140	460	charcoal	standard	Middle	Fortea and Jordá, 1976 [47]
Nerja	Vestibule8i	Beta-189081	12360	60	charcoal	AMS	Middle	Jordá and Aura, 2008 [68]
Nerja	Vestibule 8i	Ubar-157	15990	260	charcoal	standard	Middle	Jordá and Aura, 2008 [68]
Nerja	Vestibule8K+L	Ubar-158	18420	530	charcoal	standard	Middle	Jordá et al., 1990 [69]
Nerja	NV9(C4VIII)	GifA-102021	21140	190	charcoal	AMS	Lower	Jordá and Aura, 2008 [68]
Nerja	Vestibule8c	Ubar-98	17940	200	charcoal	standard	Solutreo-Gravettian?	Jordá et al., 1990 [69]
Nerja	M79/8	GAK-8965	16520	540	charcoal	standard	?	Jordá and Aura, 2008 [68]
Parpalló	4'25–4'00	Birm-521	17896	340	bone	standard	Solutreo-Gravettian	Bofinger and Davidson, 1977 [70]
Parpalló	T16	OxA-22651	19020	100	bone	AMS	Solutreo-Gravettian	Aura et al., 2012 [71]
Parpalló	7'25–6'25	Birm-520	20170	380	bone	standard	Lower	Bofinger and Davidson. 1977 [70]
Parpalló	7'25–6'25	Birm-859	20490	900	bone	standard	Lower	Bofinger and Davidson. 1977 [70]
Parpalló	5'00–4'75	Birm-861	18080	800	bone	standard	Upper	Bofinger and Davidson. 1977 [70]
Ratla del Bubo	II	Ly-5219	17360	180	charcoal	standard	Solutreo-Gravettian	Soler et al., 1990 [72]
Salemas	V.S.	ICEN-376	20250	320	bone	standard	Upper	Zilhão, 1997 [28]
Salemas	V.S	ICEN-385	19220	300	bone	standard	Upper	Zilhão, 1997 [28]
Salemas	V.S	ICEN-367	17770	420	bone	standard	Upper	Zilhão, 1997 [28]
Santa Maira	II-12	Beta-317412	19910	100	charcoal	AMS	Middle	Aura and Jordá, 2012 [33]
Vale Almoinha	5AIII	OxA-5676	19940	180	charcoal	AMS	Middle	Zilhão, 1997 [28]
Vale Almoinha	5SIII	ICEN-71	20380	150	charcoal	standard	Middle	Zilhão, 1997 [28]
Vale Boi	C4	Wk-26800	20620	160	charcoal	AMS	Upper	Cascalheira et al., 2012 [45]
Vale Boi	C	Wk-26802	20570	158	charcoal	AMS	Upper	Cascalheira et al., 2012 [45]
Vale Boi	C1	Wk-24765	19533	92	charcoal	AMS	Upper	Cascalheira et al., 2012 [45]
Vale Boi	B6	Wk-17840	18859	90	shell	AMS	Upper	Cascalheira et al., 2012 [45]
Vale Boi	B1	Wk-17840	20339	161	charcoal	AMS	Upper	Cascalheira et al., 2012 [45]
Vale Boi	Vertente2	Wk-12130	18410	165	bone	AMS	Upper	Cascalheira et al., 2012 [45]
Vale Boi	Vertente2	Wk-12131	17634	110	charcoal	AMS	Upper	Cascalheira et al., 2012 [45]

More than half of the dates (n = 38) are AMS results but very few have been submitted to the state-of-the-art sample treatment protocols, such as the ABox-SC method [73] or bone ultrafiltration. A large percentage of these dates was obtained from charcoal samples, although for most cases there was no previous species identification. Bone samples are less frequent (n = 16) but still more frequent than shells. In fact, the single dated Solutrean shell present in the database comes from layer B in Vale Boi rockshelter (Southern Portugal).

Regarding Standard Deviations (SD), only two results offer values above 1000 years while 14 dates present values below 100 years. The majority of the results presents SD values between 100 and 200 years in good agreement with what is expected from AMS results for samples within that age range.

When linked with the classical organization, most dates come from deposits whose materials indicate Upper Solutrean (n = 20) or Solutreo-Gravettian (n = 16) phases. Lower and Middle Solutrean assemblages have fewer dates, in a total of 4 and 11 results, respectively.

Not all dates were used in our analysis. In fact, given the protocol used for the Bayesian modelling (see below) we have excluded: two dates from Vale Boi’s Slope area (Wk-12130 and Wk-12131) since, for now, there is no way to check their correspondence with the rockshelter’s sequence [45]; the date from Finca Doña Martina (VERA-5101bHS), one from Ratla del Bubo (Ly-5219), three dates from Beneito (Ua-322243, Ly-3596, Ua-32244), and one from Buraca Grande (Gif-9502), for which we had no complete stratigraphic and/or assemblage characteristics information; and, finally, the single determination for Layer IV of Ambrosio (Gif-9884). This latter exclusion was related to the fact that according to Cascalheira [42] that determination can date a Proto-Solutrean component located most probably at the bottom of this layer or in the underlying Layer VI.

### Calibration and Bayesian analysis

All dates were calibrated using the IntCal13 and Marine13 curves [74] in OxCal v.4.2.4 [75]. Since the shell sample comes exclusively from the site of Vale Boi a regional ΔR value of 265±107 was used with that date.

For the Bayesian analysis we have applied, with the necessary changes, the modelling procedures used in previous works (see e.g. [73, 76, 77, 78, 79]).

Briefly, Bayesian modelling allows the incorporation, along with the calibrated likelihoods or calibrated probability distributions, of relative stratigraphic information recorded from sites [76]. We used Bayesian methods to independently model a total of 13 Solutrean sites (i.e. Ambrosio, La Boja, Bajondillo, Caldeirão, Cendres, Gorham’s, Lagar Velho, Mallaetes, Nerja, Parpalló, Salemas, Vale Almoinha and Vale Boi). Model CQL codes used for each of these sequences and respective results are provided as supporting information (Appendix A and B in S1 File). When possible, the structure of these models included more determinations from older and younger phases in the sequence in order to restrict the calibrated probability distributions of the Solutrean levels. Information regarding the stratigraphic context of these sites was collected from the available bibliography and, in certain cases, obtained by personal information with the responsible for each site.

The resolution of all models was set at 20 years and a General t-type Outlier Model [80] was used so that all dates would have a 5% prior probability of being an outlier within the sequence.

Besides performing a mathematical function of a particular set of dates, one of the most relevant returns of Bayesian modelling is the calculation (through the use of *Boundaries*) of Probability Distribution Functions (PDFs) that provide an estimate for the start and end moments of each *Phase* (set of unordered dates) or *Sequence* (set of ordered dates) at each archaeological site.

In our analysis we have used *Boundaries* to: (1) calculate the start and end PDFs of each classical-defined phase within the individual archaeological sequences; (2) determine by inserting the site *Boundaries* as *Priors* within a single *Phase* [76], the start PDFs of each Solutrean phase for Southern Iberia; (3) and define, using the *Date* command in OxCal, PDFs spanning periods of interest in the models, such as the duration of the classical-defined phases at each site.

The relation of these *Boundaries* for each site and archaeological phase were further scrutinized using the *Order* query function [75], to check for the statistical probability of one PDF to occur significantly earlier than other.

## Results

Most radiocarbon determinations present good agreement indexes within the individual stratigraphic sequences. Outliers were detected in the sites of Caldeirão, Vale Boi, Parpalló, Bajondillo and Mallaetes (see Appendix A in S1 File for complete results of the individual models) that, overall, confirm previous rejections made by the excavators of each site (e.g. [28, 45]).

**Fig 2:**
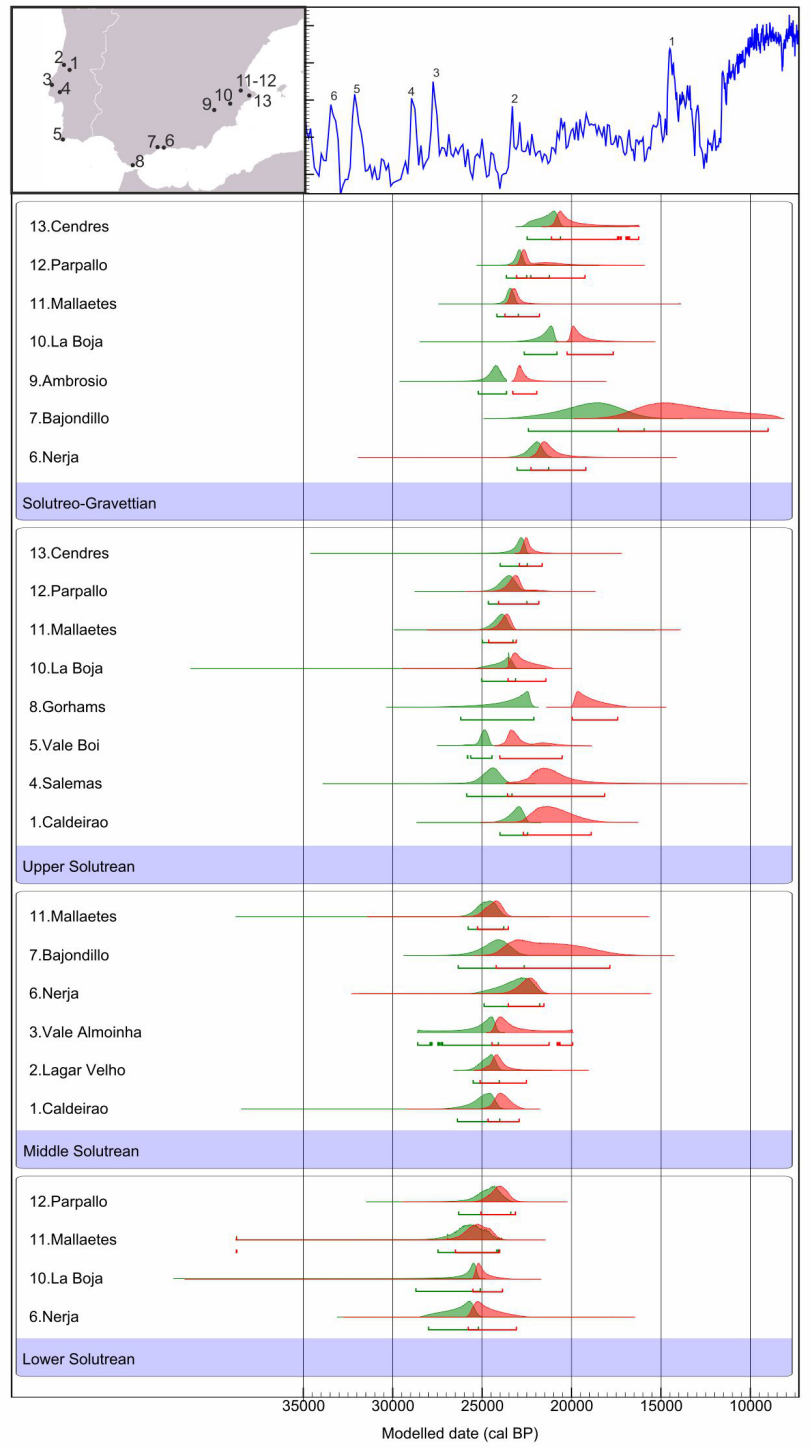
Boundary PDFs for the start (green) and end (red) of Solutrean phases at each site. Numbers correspond to the location of sites on the map. Upper part: GISP2 stable oxygen curve with numbered Greenland Interstadials. Figure plotted in OxCal v.4.2.4 [75].

Since our main goal was to test the inconsistencies of the classical chronological model in Southern Iberia we have extracted the end and start boundaries of each phase from each site model and plotted them together in Fig 2 for comparison.

The earliest evidence for the Solutrean in Southern Iberia is given by the start PDFs for the Lower Solutrean levels of La Boja, Nerja and Mallaetes, all with high probabilities of occurring shortly before c. 25 ka cal BP. When statistically ordered and compared with the remaining start PDFs these boundaries present always high chances (mostly more than 90%) of being the oldest Solutrean occupations in the region (see Appendix A in S2 File). The Lower Solutrean at Parpalló, on the contrary, seems to have begun at or slightly after the 25 ka cal BP mark.

While it was expected that, according to the traditional scheme, the oldest start boundaries, after the Lower Solutrean, would be the ones coming from Middle Solutrean levels, it is evident that the presence of at least two cases of Upper Solutrean levels (Vale Boi and Salemas) with high likelihoods to start at or slightly after 25 ka cal BP contradict the traditional scheme (Fig 2). PDFs for these sites seem, indeed, to be older or, at least, synchronous with the Middle Solutrean levels of Caldeirão, Mallaetes, Lagar Velho, Vale Almoinha and Bajondillo, and with the starting moment of the Lower Solutrean at Parpalló.

To closely examine the temporal relationship between all these start boundaries occurring around the 25 ka cal BP mark, a statistical comparison of the start PDFs of the various sites was performed (see S2 File). The results confirm that the generated age estimate for the start of the Upper Solutrean at Vale Boi have high probabilities of being older (at 95.4% probability) than the Middle Solutrean start boundaries of Bajondillo (80% chances), Lagar Velho (70% chances) and Mallaetes (60% chances) and to have 50% possibilities to be older than the Middle Solutrean start PDF from Caldeirão cave. The comparisons between the start PDF for Salemas and the remaining Middle Solutrean occupations, on the other hand, suggest that the beginning of the Upper Solutrean at this site may have occurred earlier than at Nerja (90% chances) and Bajondillo (60% chances).

Moreover, the strikingly early result given by the start PDF of the Solutreo-Gravettian phase at Ambrosio is also pertinent in this context. With high probability of occurring immediately after 25 ka cal BP it clearly indicates that the assemblages attributed to the Upper Solutrean at this site (Layer IV), stratigraphically located below this date, must have been deposited roughly at the same time or even earlier than the Vale Boi and Salemas Solutrean levels. Statistical ordering also seems to corroborate the fact that the Ambrosio’s Solutreo-Gravettian start PDF have high chances to occur earlier than all the Upper Solutrean (with exception of Vale Boi and Salemas) and even earlier (90% chance at 95.4% probability) than the Middle Solutrean start moment in Nerja.

**Fig 3:**
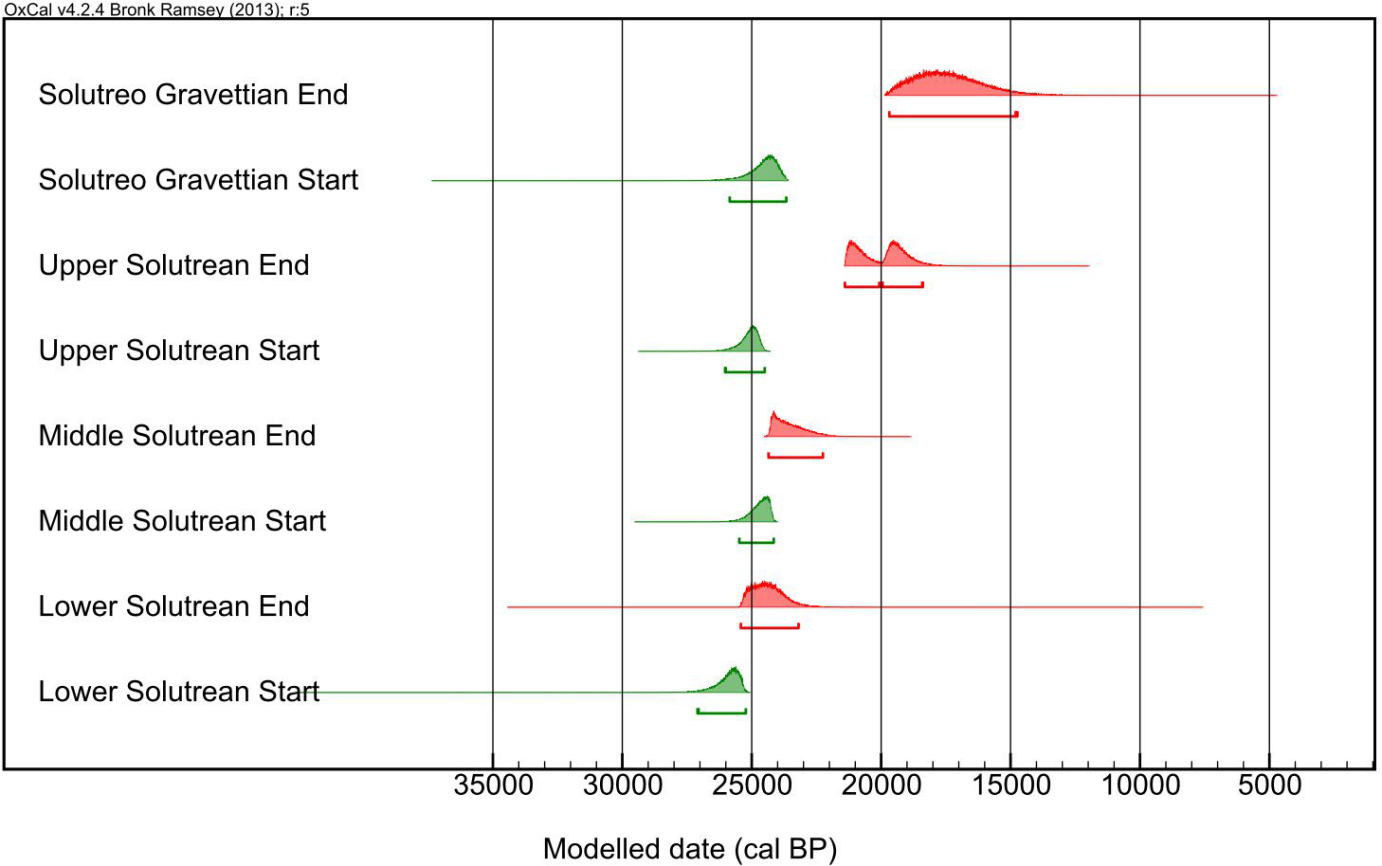
Bayesian modelled PDFs for the start (green) and end (red) *Boundaries* of the each classical Solutrean phase in Southern Iberia. These *Priors* were calculated combining the *Boundaries* for each moment at each site into a single *Phase* model. Divergences detected between the end of the Middle Solutrean in this figure and the late *Boundaries* for Bajondillo and Nerja (Fig 2) indicate that when compared to the remaining sites, at the 95.4% probability range, the most probable limit is at 24,350–22,248 cal BP.

When combined into a single phase Bayesian model for the whole region, the start PDFs for the Solutrean in Southern Iberia range (at 95.4% probability) from 27,095–25,228 cal BP for the Lower Solutrean, 25,477–24,150 for the Middle Solutrean, 26,035–24,497 for the Upper Solutrean, and 25,867–23,650 for the Solutreo-Gravettian (Fig 3).

The results clearly indicate that, in general, the traditional succession scheme for the Lower, Middle, Upper and Solutreo-Gravettian phases is not confirmed with the currently available radiocarbon dataset using Southern Iberia as a whole. In fact, the radiocarbon Bayesian results unequivocally contradicts the traditional succession for the beginning of each phase, suggesting that the Upper Solutrean started 5 centuries earlier than the Middle Solutrean; and the Solutreo-Gravettian in Mediterranean Spain appeared only 200 years or 3 centuries before the Middle Solutrean.

In contrast, the plot of the end boundaries for each phase (Fig 3) partially corroborates the traditional perspective that each phase ends progressively, in the traditional ordering, at different times. Thus, the end of the Lower Solutrean most probably took place between 25,421–23,153 cal BP, the Middle Solutrean between 24,352–22,234 cal BP, the Upper Solutrean between 21,400–18,375 cal BP, and the Solutreo-Gravettian, with the largest span, between 19,684–14,701 cal BP. Some overlap between this ranges is evident but statistical comparison seems to, largely, confirm a gradual ending sequence (see Appendix B in S2 File).

Distribution by sites shows that the Lower and Middle Solutrean ending PDFs present, generically, a good agreement across sites, while Upper and Solutreo-Gravettian are more inconsistent. In this context the most notable occurrences are the persistence of the Solutreo-Gravettian at Bajondillo until well after the conclusion of the remaining phases at all sites, as well as the almost synchronous end of the Upper Solutrean of Gorham’s cave and the Solutreo-Gravettian at La Boja (Fig 2).

## Discussion

The results just presented have broad implications for the traditional perspectives on the LGM adaptations in Southern Iberia. The overwhelming impression from our analysis is, in effect, one of inconsistency across sites and within regions, with the starting moments of the traditionally-defined phases of the Solutrean techno-complex not falling in the same order as previously assumed from stratigraphic records of some of the key-sequences.

The only phase that seems to consistently occur at an earlier time than the others is the Lower Solutrean, though again, this phase seems to exist only in Mediterranean Spain. This pattern is attested by the results from Nerja (level 9), Mallaetes (level VI) and La Boja (level E).

Some caution is, however, necessary when attributing these levels to a Lower Solutrean stage, as defined in the classical scheme. Recent data on the lithic assemblages from Level 9 at Nerja revealed, for example, the presence of a small component of Proto-Solutrean index technology, the Vale Comprido point/blank, together with some pointes à face plan [33, 43]. This causes, in our opinion, a questionable attribution of those horizons to the traditional Lower Solutrean phase. The same holds true for the case of Mallaetes, where a small set of pointes à face plan have been identified by Fortea and Jordá [47]; the authors clearly state that most of them are hard to classify as typical implements, falling somewhere between Smith’s [25] subtype E and blades pointed by marginal/oblique retouch.

At La Boja rockshelter the attribution of Layer E to the classical Lower Solutrean also does not have, so far, a strong techno-typological support [57]. The material coming from that layer is, indeed, very small in quantity, with only one artifact unquestionable classified as a pointe à face plan, and thus we cannot exclude sampling problems in the attribution.

Overall, even though the dates obtained in our model are coincident, for example, with the results available for the Lower Solutrean levels at the Laugerie-Haute and Les Peyrugues sequences (dated to c. 25.5–23.7 ka cal. BP) in Southwestern France [10, 81, 82, 83], the insecurity in the attribution of the Southern Iberia assemblages is, unfortunately, for now, not enough for a safe identification and characterization of a Lower Solutrean phase in the region.

**Fig 4:**
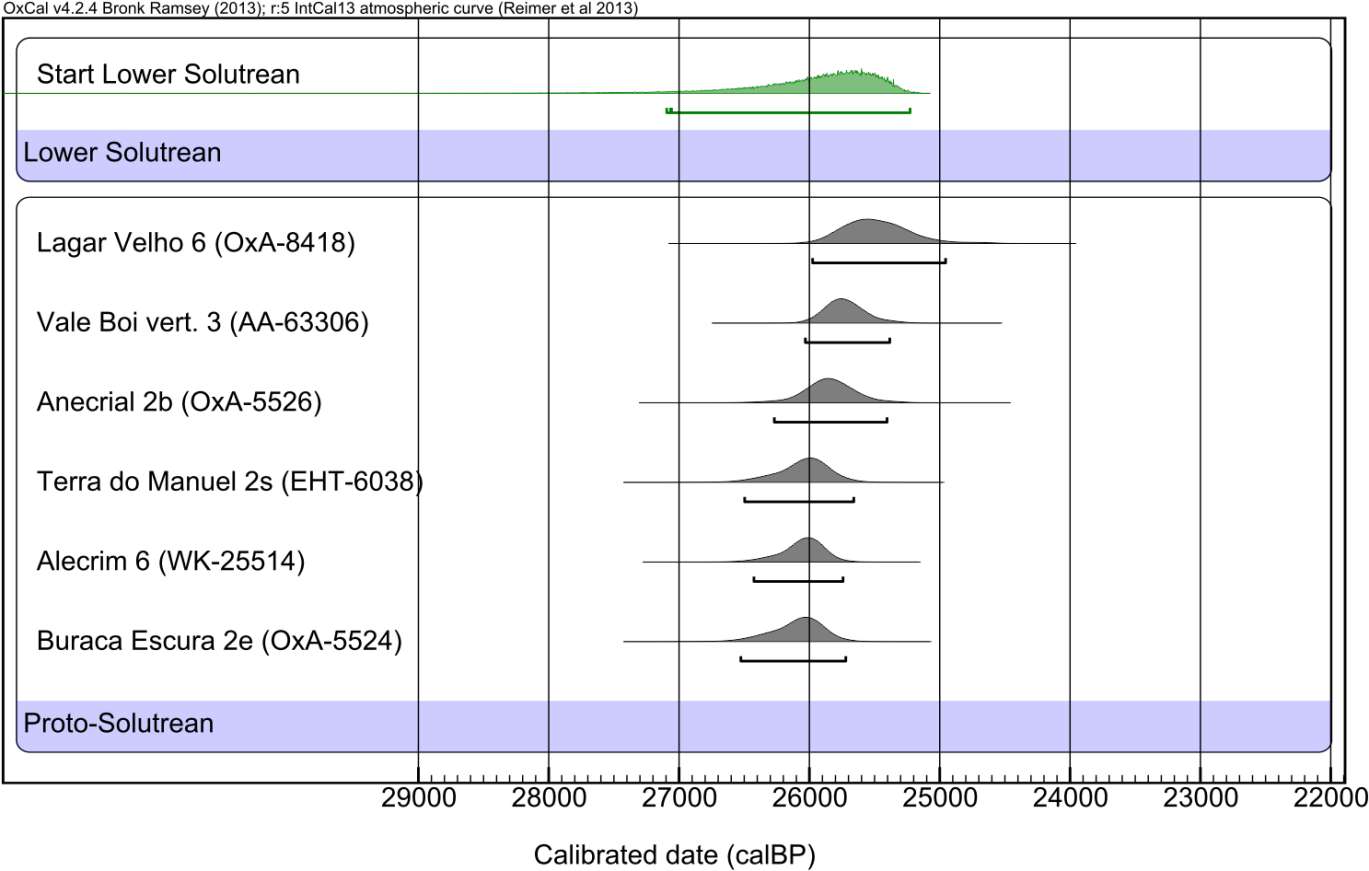
Comparison of some of the available dates for the Proto-Solutrean in Western Iberia [84] with the *Boundary* PDF for the start of the Lower Solutrean in Southern Iberia.

Further, when compared with some of the available dates for the Proto-Solutrean in Central and Southern Portugal [84] (Fig 4), the start boundary defined within our analysis for the Lower Solutrean exhibit high probabilities of occurring at the same time or even at an earlier moment. This perspective has been corroborated by Fortea and Jordá [47] and Aura et al [43] for the sites of Mallaetes and Nerja, respectively. Thus, the Lower Solutrean may, in fact, be nothing else other than a Proto-Solutrean horizon, that was not recognized as such since this phase had not been defined when those sites were excavated and the lithic assemblages were analyzed.

More secure seems, in contrast, the occurrence of contexts with stemmed and shouldered projectiles, traditionally attributed to the Upper Solutrean or Solutreo-Gravettian, at between one to two millennia earlier than previously thought. Thus, the Upper Solutrean is either in clear synchrony or occurring slightly earlier than the onset of most Middle Solutrean contexts in Southern Iberia.

Vale Boi is, in this perspective, the most illustrative example, since it is one of the most recently excavated contexts and from where an in-depth lithic techno-typological analysis of the entire sequence is available [42, 45, 59]. Lithic data allows to clearly contradict any arguments that attempt to attribute the earliest radiocarbon results to the existence of post-depositional processes at Vale Boi. The absence of pointes à face plan in the Vale Boi rockshelter sequence indicates the inexistence of a Lower or Middle Solutrean occupation as traditionally defined [42, 59]. The only element recently published as possibly identical, in the style of its representations, to the elements attributable to the Middle Solutrean in the Valencia region (e.g. Parpalló [85]) was a small engraved slab recovered from the oldest Solutrean horizons of Vale Boi rockshelter [85, 86]. This element does not confirm, however, the presence of a Middle Solutrean phase at that site but only reinforces, through an independent variable, the chronological agreement between archaeological layers.

The divergences detected between the traditional organization of the Solutrean in Southern Iberia and the available radiocarbon database used in our analysis might be explained by a set of different factors that are important to address here.

**Fig 5:**
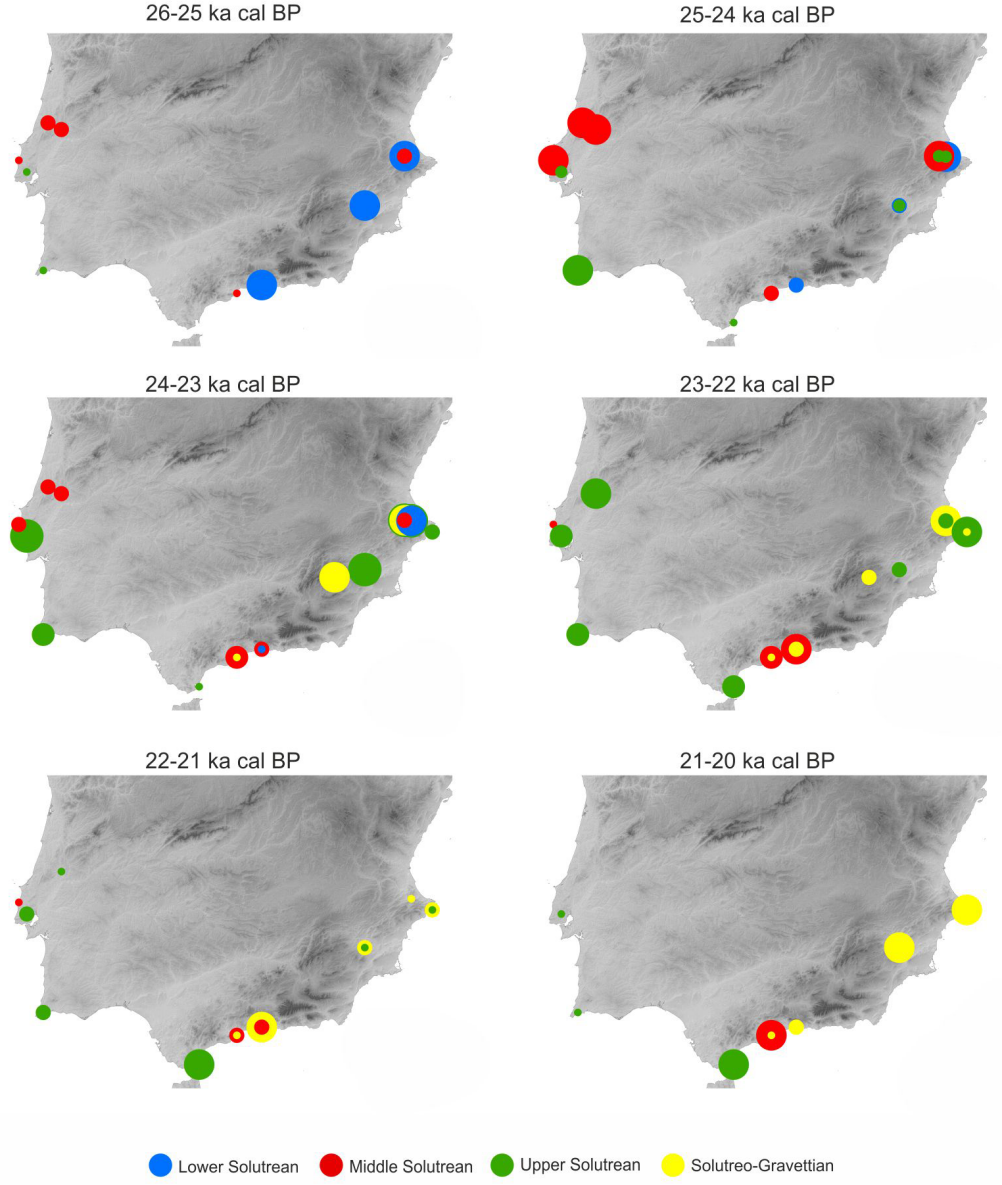
Time slices for Southern Iberia between 26 and 20 ka cal BP showing the distribution of modelled ages of the classical Solutrean phases. The size of the dots represents increasing and decreasing levels of the 95.4% probability ranges determined from the duration (date range) of each phase, as calculated by individual Bayesian site models (see Appendix A in S1 File). Dots with two colors indicate overlapping date range probabilities for two or more phases found at the same site.

As a first hypothesis we wanted to check the possible existence of regional discrepancies in the timing for the development of the various Solutrean phases in the different regions. To test this we mapped the spatiotemporal relationship between the four phases during the period c. 26–20 ka cal BP, using 1000 years’ time slices (Fig 5).

As foreseen from the numeric results, no specific pattern seem to emerge from the distribution across and within regions, with the highly probable coexistence of a great diversity of point types at most stages within this time-span. Nevertheless, this pattern could have occurred regardless the fact that, at some sites, the classical-defined Lower and Middle Solutrean assemblages predate the Upper and Solutreo-Gravettian levels stratigraphically where they co-occur.

Although Southern Portugal seems to present highest probabilities for the occurrence of the earliest pedunculated projectiles (25–24 ka cal BP), we should bear in mind the case of Ambrosio where these type of points are clearly identified in a level that has necessarily been deposited between c. 26 ka cal BP and 24 ka cal BP (according to the results presented above).

Two clear tendencies can be outlined related to the distribution patterns of the Lower Solutrean and Solutreo-Gravettian type assemblages. In fact, these two components seem to be restricted to the Mediterranean region and totally absent from the Atlantic facade. In the first case, this pattern may be masked by the uncertainty in the recognition of a classical Lower Solutrean phase in these sites, as mentioned above, since this may in fact represent Proto-Solutrean occupations at those sites, contemporaneous with the Proto-Solutrean in the other regions of southern Iberia. In the second case, however, the presence of Solutreo-Gravettian assemblages in Central and Southern Portugal is, indeed, yet to be confirmed, despite the putative attribution of some assemblages by Zilhão [11, 28]. From the 22–21 ka cal BP slice onwards there is a considerable decrease in the probability of occurrence of any of the classic phases in Southern and Central Portugal, after which the Magdalenian is clearly established in Portugal [87].

The second hypothesis is the one related with the quality and resolution of the current available radiocarbon database. As mentioned above, some of the dates used in our modelling were obtained long ago, when the current sample treatment protocols were not available. Previous underestimates for the real age of specific dates have been proved by recent radiocarbon dating programs using modern cleaning and decontamination processes [73, 88, 89] and, thus, we cannot exclude similar problems to occur with the Solutrean data.

In the case of Southern Iberia, the most notorious example of problems associated with the past radiocarbon dates comes from the Ambrosio rockshelter, where dates obtained from samples collected during the early 20^th^ century proved, recently, to be very young when compared with the results of new AMS dated samples [60]. The difference is of c. 4 to 5 thousand years, with the early dates of the deposit setting the occupations around 19 ka cal BP and the latest results showing, for the same level and tested with multiple samples, an average age of c. 24 ka cal BP.

Together with the Vale Boi data, the starting PDF from Ambrosio’s Solutreo-Gravettian layer is one of the important outcomes of our model that raises serious questions on the validity of the global application of the traditional chrono-stratigraphic perspective. The two sites were, contrarily to sites such as Parpalló, Mallaetes or Caldeirão caves, dated very recently, with access to new procedures, and, moreover, with abundant sets of dates for each stratigraphic unit.

In this perspective, forcing the existence of a Lower Solutrean to Solutreo-Gravettian chronological succession as previously argued would, in theory, ask for the careful consideration of dates obtained some time ago. This would mean that dates from the Middle Solutrean of several sites had necessarily to be older than the earliest Upper Solutrean and Solutreo-Gravettian layers of Vale Boi, Ambrosio and Salemas which, in turn, will leave very little space between the Lower (and the Proto-Solutrean) and the Upper Solutrean to accommodate a Middle Solutrean phase. Unfortunately, as previously noted by Zilhão [11], if existent, such a narrow timeframe will hardly ever be recognized by the current resolution of absolute dating methods.

The third and final factor that may have a great influence in the differences seen now is the lack of systematics in the classification of the different phases across sites and regions associated with the frequent stratigraphical problems at some of the so-called key-sites. In addition there are also sampling problems that might be associated with the excavation of small areas at some of these sites, likely creating false-negative results for the presence of specific point types. One good example in this context is the classification of the Middle Solutrean layers at Parpalló. The attribution of levels to the Middle Solutrean (i.e., the artificial levels between 6,25–5,25 meters) at this cave is based on a dominant presence of laurel-leaves and their stratigraphic position below levels with large amounts of stemmed and shouldered projectiles. What is often neglected is, however, the presence of a set of these latter types amongst the lower levels (Fig 37 in [1]). In any case, their occurrence can either clearly drag the time of appearance of Upper Solutrean type-fossils to earlier chronologies, or indicate serious problems associated with the interpretation of the available stratigraphic information (see [9] for more details).

## Conclusions

The present study shows solid evidence against the existence of a chronological succession of Solutrean phases as defined by the traditional model, borrowed from the French reality and applied to Southern Iberia, by several authors, over time. Our results corroborate, in part, Straus’ [14, 23, 35, 90, 91, 92] perspective for the Solutrean in Northern Iberia, where, according to the author, point types traditionally considered to be recent in age do also appear in early contexts across the region. The author explained this pattern linking different tool kits to different classes of game animals and site surroundings, demonstrating the existence of functional variants within the Vasco-Cantabrian Solutrean.

Of course that, similarly to Cantabria, the differences between assemblages in the relative frequencies of the various types of points could correspond either to dissimilarities in the hunted species or to preferred hunting methods, or they could also result from replacement trends not occurring in the exact same mode or at the same time in the different Solutrean territories [14]. However, in the case of Southern Iberia, the possible meaning or underlying organization for the use of the different point types and the patterns of their presence and/or absence across sites is not yet clear. In-depth and systematic studies of the lithic industries and faunal assemblages, as well as a comprehensive dating program for the Solutrean across all regions are imperative and will certainly help to clarify this and other questions.

Still, despite the possible existence of misleading factors among the currently available dataset, the main impacts of our analysis on the current knowledge of the LGM adaptations in Southern Iberia can be summarized as follow:

1. The call into doubt of the status of the traditionally-defined type-fossils as precise temporal markers for each Solutrean phase in Southern Iberia;
2. The confirmation of the presence of tanged “Parpalló-type” points at a much earlier time (c. 25 ka cal BP) than previously thought;
3. The potential contemporaneity at a very early moment (c. 25 ka cal BP) of the so-called Middle and Upper Solutrean/Solutreo-Gravettian phases (and thus should preferably be called facies)
4. The likely organization, from a broad chrono-cultural point of view, of the adaptive systems surrounding the LGM event in just two discrete contiguous entities, known as the Proto-Solutrean and the Solutrean.

## Supporting Information

S1 File Bayesian analysis data. Modelling results for each archaeological site (**Appendix A**); Bayesian CQL Codes (**Appendix B**)**.**
(DOCX)

S2 File Statistical results of the application of the *Order* query function. Start boundaries (**Appendix A**). End boundaries (**Appendix B**).
(XLSX)
