# Supplementary material for: On the Chronological Structure of the Solutrean in Southern Iberia
Source: PLoS One. 2015 Sep 10;10(9):e0137308. doi: 10.1371/journal.pone.0137308 (PMC4565679; doi:10.1371/journal.pone.0137308)
Supplement: S1 File — Modelling results for each archaeological site (Appendix A); Bayesian CQL Codes (Appendix B). (DOCX) [file pone.0137308.s001.docx]

**S1 Supporting Information**

## On the chronological structure of the Solutrean in Southern Iberia

**Appendix A - Bayesian modelling results for each archaeological site**

**Central Portugal**

**Caldeirão**

**
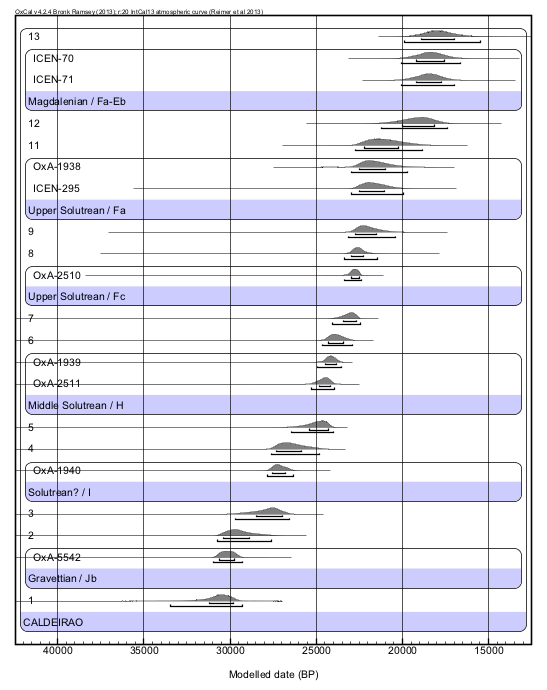
**

**Lagar Velho**

**
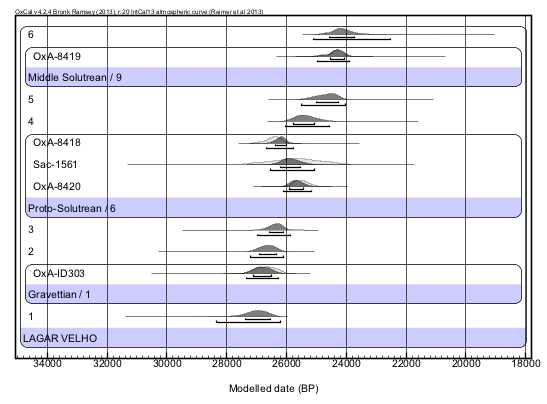
**

**Vale Almoinha**

**
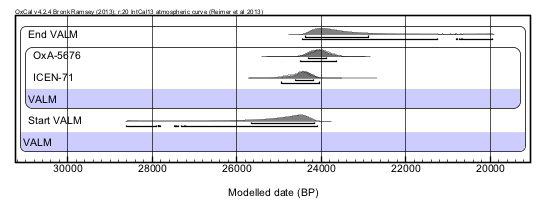
**

**Salemas**

**
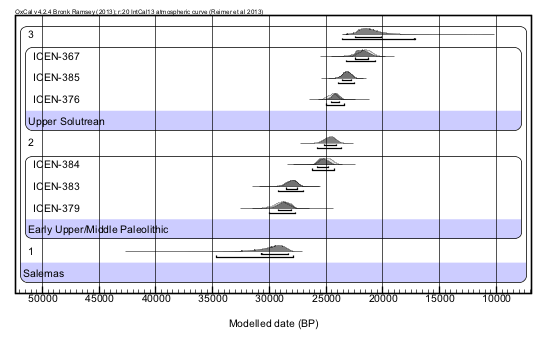
**

**Southern Portugal**

**Vale Boi
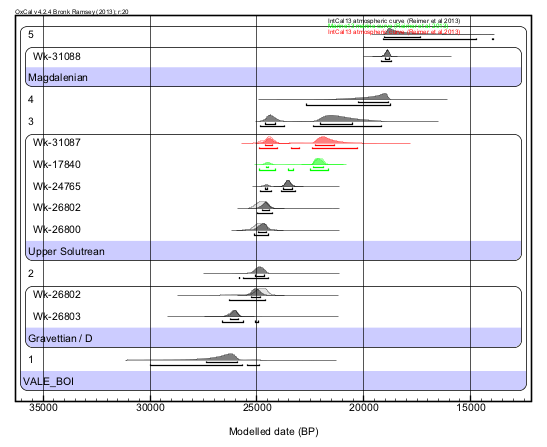
**

**Southwestern Spain**

**Gorham’s**

**
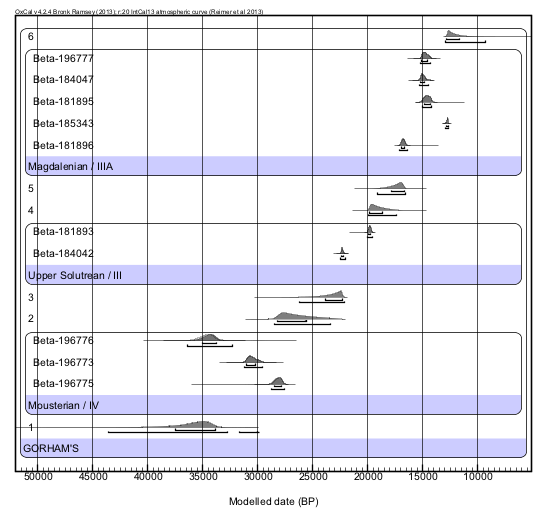
**

**Nerja**

**
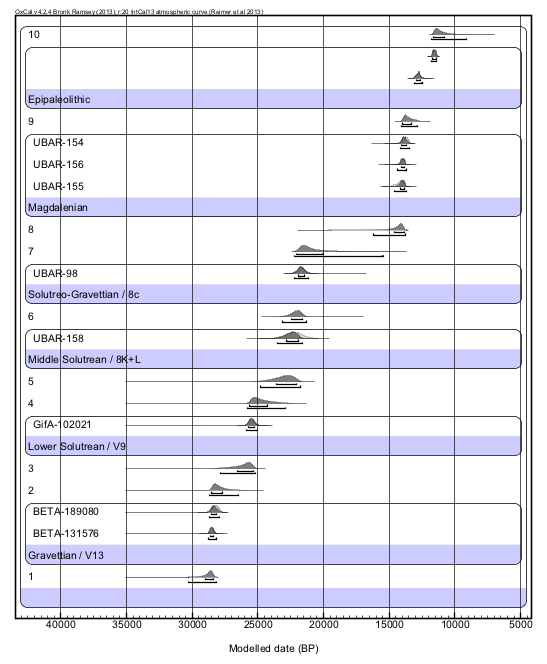
**

**Bajondillo**

**
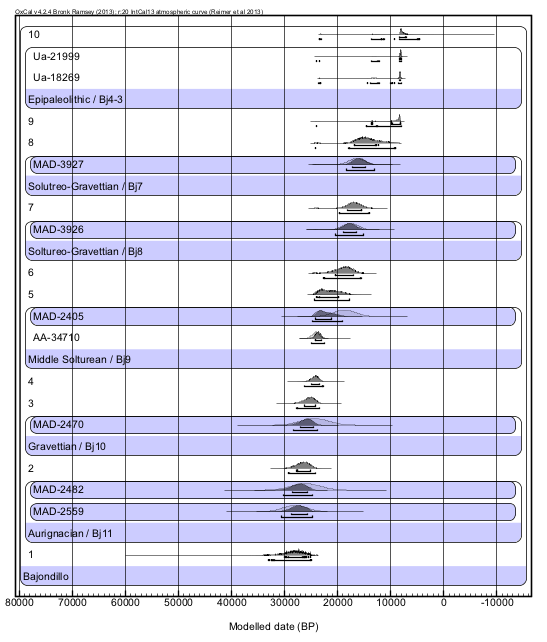
**

**Southeastern Spain**

**Ambrosio**

**
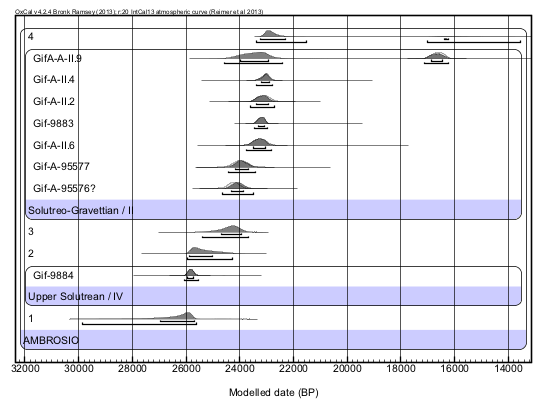
**

**La Boja**

**
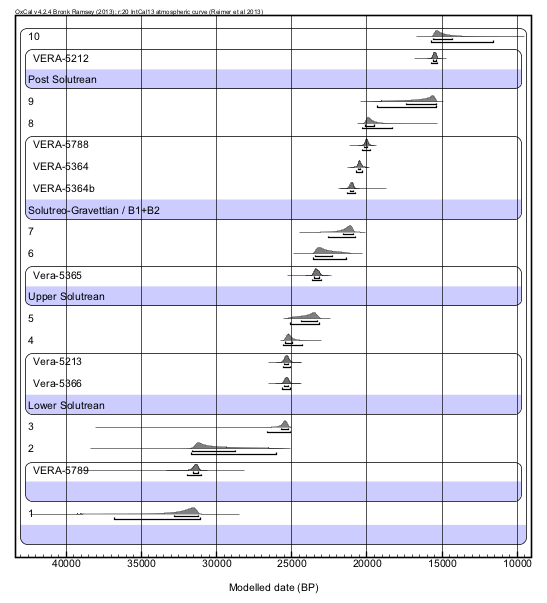
**

**Mallaetes**

**
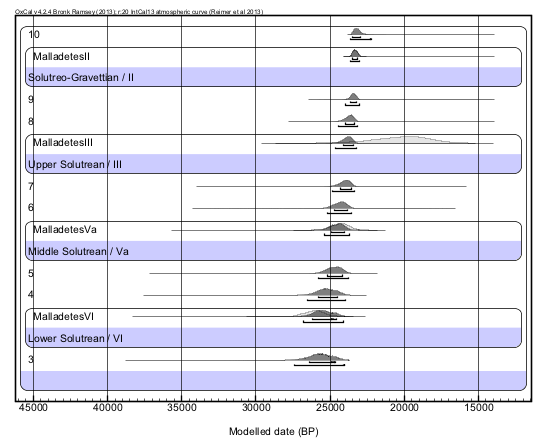
**

**Parpalló**

**
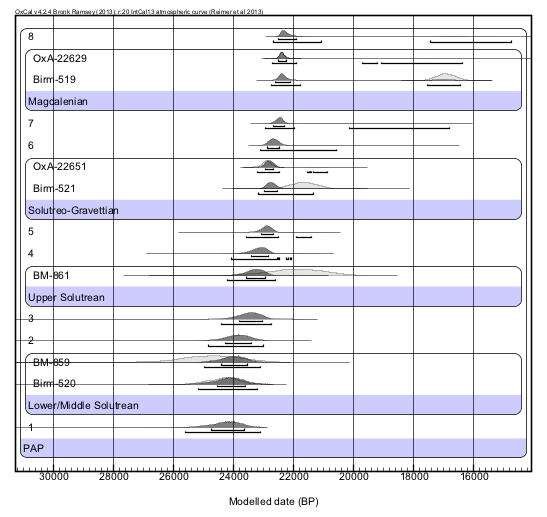
**

**Cendres**

**
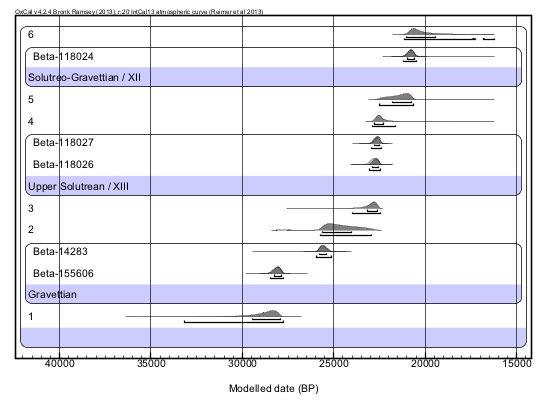
**

**S1B - Bayesian CQL Codes**

**Central Portugal**

**Caldeirão**

Options()

{

Resolution=20;

};

Plot()

{

Outlier_Model("General",T(5),U(0,4),"t");

Sequence("CALDEIRAO")

{

Boundary("1");

Phase("Gravettian / Jb")

{

R_Date("OxA-5542", 26020, 320)

{

Outlier("General", 0.05);

};

};

Boundary("2");

Boundary("3");

Phase("Solutrean? / I")

{

R_Date("OxA-1940", 22900, 380)

{

Outlier("General", 0.05);

};

};

Boundary("4");

Boundary("5");

Phase("Middle Solutrean / H")

{

R_Date("OxA-2511", 20530, 270)

{

Outlier("General", 0.05);

};

R_Date("OxA-1939", 19900, 260)

{

Outlier("General", 0.05);

};

};

Boundary("6");

Boundary("7");

Phase("Upper Solutrean / Fc")

{

R_Date("OxA-2510", 18840, 200)

{

Outlier("General", 0.05);

};

};

Boundary("8");

Boundary("9");

Phase("Upper Solutrean / Fa")

{

R_Date("ICEN-295", 21200, 2300)

{

Outlier("General", 0.05);

};

R_Date("OxA-1938", 20400, 270)

{

Outlier("General", 0.05);

};

};

Boundary("11");

Boundary("12");

Phase("Magdalenian / Fa-Eb")

{

R_Date("ICEN-71", 15170, 740)

{

Outlier("General", 0.05);

};

R_Date("ICEN-70", 14450, 890)

{

Outlier("General", 0.05);

};

};

Boundary("13");

};

Sequence()

{

Boundary("=5");

Date("Caldeirao Middle Solutrean");

Boundary("=6");

};

Sequence()

{

Boundary("=7");

Date("Caldeirao Upper Solutrean");

Boundary("=8");

};

Sequence("Total")

{

Boundary("=5");

Date("Caldeirao");

Boundary("=8");

};

};

**Lagar Velho**

Options()

{

Resolution=20;

};

Plot()

{

Outlier_Model("General",T(5),U(0,4),"t");

Sequence("LAGAR VELHO")

{

Boundary("1");

Phase("Gravettian / 1")

{

R_Date("OxA-ID303", 22390, 280)

{

Outlier("General", 0.05);

};

};

Boundary("2");

Boundary("3");

Phase("Proto-Solutrean / 6")

{

R_Date("OxA-8420", 21180, 240)

{

Outlier("General", 0.05);

};

R_Date("Sac-1561", 21380, 810)

{

Outlier("General", 0.05);

};

R_Date("OxA-8418", 22180, 180)

{

Outlier("General", 0.05);

};

};

Boundary("4");

Boundary("5");

Phase("Middle Solutrean / 9")

{

R_Date("OxA-8419", 20200, 180)

{

Outlier("General", 0.05);

};

};

Boundary("6");

};

Sequence()

{

Boundary("=5");

Date("Lagar Velho Middle Solutrean");

Boundary("=6");

};

};

**Vale Almoinha**

Options()

{

Resolution=20;

};

Plot()

{

Outlier_Model("General",T(5),U(0,4),"t");

Sequence("VALM")

{

Boundary("Start VALM");

Phase("VALM")

{

R_Date("ICEN-71", 20380, 150)

{

Outlier("General", 0.05);

};

R_Date("OxA-5676", 19940, 180)

{

Outlier("General", 0.05);

};

};

Boundary("End VALM");

};

Sequence()

{

Boundary("=Start VALM");

Date("VALM");

Boundary("=End VALM");

};

};

**Salemas**

Options()

{

Resolution=20;

SD2=TRUE;

};

Plot()

{

Outlier_Model("General",T(5),U(0,4),"t");

Sequence("Salemas")

{

Boundary("1");

Phase("Early Upper/Middle Paleolithic")

{

R_Date("ICEN-379", 24820, 550)

{

Outlier("General", 0.05);

};

R_Date("ICEN-383", 23830, 580)

{

Outlier("General", 0.05);

};

R_Date("ICEN-384", 20740, 470)

{

Outlier("General", 0.05);

};

};

Boundary("2");

Phase("Upper Solutrean")

{

R_Date("ICEN-376", 20250, 320)

{

Outlier("General", 0.05);

};

R_Date("ICEN-385", 19220, 300)

{

Outlier("General", 0.05);

};

R_Date("ICEN-367", 17770, 420)

{

Outlier("General", 0.05);

};

};

Boundary("3");

};

Sequence()

{

Boundary("=2");

Date("Salemas Upper Solutrean");

Boundary("=3");

};

};

**Southern Portugal**

**Vale Boi**

Options()

{

Resolution=20;

};

Plot()

{

Outlier_Model("General",T(5),U(0,4),"t");

Sequence(VALE_BOI)

{

Boundary("1");

Phase("Gravettian / D")

{

R_Date("Wk-26803", 21859, 186)

{

Outlier("General", 0.05);

};

R_Date("Wk-26802", 20570, 158)

{

Outlier("General", 0.05);

};

};

Boundary("2");

Sequence("Upper Solutrean")

{

R_Date("Wk-26800", 20620, 160)

{

Outlier("General", 0.05);

};

R_Date("Wk-26802", 20570, 158)

{

Outlier("General", 0.05);

};

R_Date("Wk-24765", 19533, 92)

{

Outlier("General", 0.05);

};

Curve("Marine13","Marine13.14c");

Delta_R("LocalMarine",265,107);

R_Date("Wk-17840", 18859, 90)

{

Outlier("General", 0.05);

};

Curve("IntCal13","IntCal13.14c");

R_Date("Wk-31087", 20339, 161)

{

Outlier("General", 0.05);

};

};

Boundary("3");

Boundary("4");

Phase("Magdalenian")

{

R_Date("Wk-31088", 15660, 86)

{

Outlier("General", 0.05);

};

};

Boundary("5");

};

Sequence()

{

Boundary("=2");

Date("Vale Boi Upper Solutrean");

Boundary("=3");

};

};

**Southwestern Spain**

**Gorham’s**

Options()

{

Resolution=20;

};

Plot()

{

Outlier_Model("General",T(5),U(0,4),"t");

Sequence("GORHAM'S")

{

Boundary("1");

Phase("Mousterian / IV")

{

R_Date("Beta-196775", 24010, 320)

{

Outlier("General", 0.05);

};

R_Date("Beta-196773", 26400, 440)

{

Outlier("General", 0.05);

};

R_Date("Beta-196776", 30560, 720)

{

Outlier("General", 0.05);

};

};

Boundary("2");

Boundary("3");

Phase("Upper Solutrean / III")

{

R_Date("Beta-184042", 18440, 80)

{

Outlier("General", 0.05);

};

R_Date("Beta-181893", 16420, 60)

{

Outlier("General", 0.05);

};

};

Boundary("4");

Boundary("5");

Phase("Magdalenian / IIIA")

{

R_Date("Beta-181896", 13870, 80)

{

Outlier("General", 0.05);

};

R_Date("Beta-185343", 10880, 80)

{

Outlier("General", 0.05);

};

R_Date("Beta-181895", 12460, 100)

{

Outlier("General", 0.05);

};

R_Date("Beta-184047", 12640, 100)

{

Outlier("General", 0.05);

};

R_Date("Beta-196777", 12540, 100)

{

Outlier("General", 0.05);

};

};

Boundary("6");

};

Sequence()

{

Boundary("=3");

Date("Gorhams Upper Solutrean");

Boundary("=4");

};

};

**Nerja**

Options()

{

Resolution=20;

};

Plot()

{

Outlier_Model("General",T(5),U(0,4),"t");

Sequence()

{

Boundary("1");

Phase("Gravettian / V13")

{

R_Date("BETA-131576", 24480, 110)

{

Outlier("General", 0.05);

};

R_Date("BETA-189080", 24200, 200)

{

Outlier("General", 0.05);

};

};

Boundary("2");

Boundary("3");

Phase("Lower Solutrean / V9")

{

R_Date("GifA-102021", 21140, 190)

{

Outlier("General", 0.05);

};

};

Boundary("4");

Boundary("5");

Phase("Middle Solutrean / 8K+L")

{

R_Date("UBAR-158", 18420, 530)

{

Outlier("General", 0.05);

};

};

Boundary("6");

Phase("Solutreo-Gravettian / 8c")

{

R_Date("UBAR-98", 17940, 200)

{

Outlier("General", 0.05);

};

};

Boundary("7");

Boundary("8");

Phase("Magdalenian")

{

R_Date("UBAR-155", 12190, 150)

{

Outlier("General", 0.05);

};

R_Date("UBAR-156", 12130, 130)

{

Outlier("General", 0.05);

};

R_Date("UBAR-154", 11930, 160)

{

Outlier("General", 0.05);

};

};

Boundary("9");

Phase("Epipaleolithic")

{

R_Date(10860, 160)

{

Outlier("General", 0,05);

};

R_Date(10040, 40)

{

Outlier("General", 0,05);

};

};

Boundary("10");

};

Sequence()

{

Boundary("=3");

Date("Nerja Lower Solutrean");

Boundary("=4");

};

Sequence()

{

Boundary("=5");

Date("Nerja Middle Solutrean");

Boundary("=6");

};

Sequence()

{

Boundary("=6");

Date("Nerja Solutreo-Gravettian");

Boundary("=7");

};

Sequence("Total")

{

Boundary("=3");

Date("Nerja");

Boundary("=7");

};

};

**Bajondillo**

Options()

{

Resolution=20;

};

Plot()

{

Outlier_Model("General",T(5),U(0,4),"t");

Sequence("Bajondillo")

{

Boundary("1");

Phase("Aurignacian / Bj11")

{

Age("MAD-2559", N(28019, 2334))

{

Outlier("General", 0.05);

};

Age("MAD-2482", N(26013, 2777))

{

Outlier("General", 0.05);

};

};

Boundary("2");

Phase("Gravettian / Bj10")

{

Age("MAD-2470", N(24344, 2653))

{

Outlier("General", 0.05);

};

};

Boundary("3");

Boundary("4");

Phase("Middle Solturean / Bj9")

{

R_Date("AA-34710", 19990, 480)

{

Outlier("General", 0.05);

};

Age("MAD-2405", N(18701, 2154))

{

Outlier("General", 0.05);

};

};

Boundary("5");

Boundary("6");

Phase("Soltureo-Gravettian / Bj8")

{

Age("MAD-3926", N(17582, 1521))

{

Outlier("General", 0.05);

};

};

Boundary("7");

Phase("Solutreo-Gravettian / Bj7")

{

Age("MAD-3927", N(16438, 1497))

{

Outlier("General", 0.05);

};

};

Boundary("8");

Boundary("9");

Phase("Epipaleolithic / Bj4-3")

{

R_Date("Ua-18269", 7475, 80)

{

Outlier("General", 0.05);

};

R_Date("Ua-21999", 7325, 65)

{

Outlier("General", 0.05);

};

};

Boundary("10");

};

Sequence()

{

Boundary("=4");

Date("Bajondillo Middle Solutrean");

Boundary("=5");

};

Sequence()

{

Boundary("=6");

Date("Bajondillo Solutreo-Gravettian");

Boundary("=8");

};

Sequence("Total")

{

Boundary("=4");

Date("Bajondillo");

Boundary("=8");

};

};

**Southeastern Spain**

**Ambrosio**

Options()

{

Resolution=20;

};

Plot()

{

Outlier_Model("General",T(5),U(0,4),"t");

Sequence("AMBROSIO")

{

Boundary("1");

Phase("Upper Solutrean / IV")

{

R_Date("Gif-9884", 21520, 120)

{

Outlier("General", 0.05);

};

};

Boundary("2");

Boundary("3");

Phase("Solutreo-Gravettian / II")

{

R_Date("Gif-A-95576?", 20150, 200)

{

Outlier("General", 0.05);

};

R_Date("Gif-A-95577", 19950, 210)

{

Outlier("General", 0.05);

};

R_Date("Gif-A-II.6", 19300, 190)

{

Outlier("General", 0.05);

};

R_Date("Gif-9883", 19250, 70)

{

Outlier("General", 0.05);

};

R_Date("Gif-A-II.2", 19170, 190)

{

Outlier("General", 0.05);

};

R_Date("Gif-A-II.4", 19110, 90)

{

Outlier("General", 0.05);

};

R_Date("GifA-A-II.9", 13740, 140)

{

Outlier("General", 0.05);

};

};

Boundary("4");

};

Sequence()

{

Boundary("=1");

Date("Upper Solutrean");

Boundary("=2");

};

Sequence()

{

Boundary("=3");

Date("Solutreo-Gravettian");

Boundary("=4");

};

};

**La Boja**

Options()

{

Resolution=20;

};

Plot()

{

Outlier_Model("General",T(5),U(0,4),"t");

Sequence()

{

Boundary("1");

Phase("")

{

R_Date("VERA-5789", 27620, 230)

{

Outlier("General", 0.05);

};

};

Boundary("2");

Boundary("3");

Phase("Lower Solutrean")

{

R_Date("Vera-5366", 20980, 120)

{

Outlier("General", 0.05);

};

R_Date("Vera-5213", 20980, 110)

{

Outlier("General", 0.05);

};

};

Boundary("4");

Boundary("5");

Phase("Upper Solutrean")

{

R_Date("Vera-5365", 19390, 100)

{

Outlier("General", 0.05);

};

};

Boundary("6");

Boundary("7");

Phase("Solutreo-Gravettian / B1+B2")

{

R_Date("VERA-5364b", 17430, 70)

{

Outlier("General", 0.05);

};

R_Date("VERA-5364", 16990, 70)

{

Outlier("General", 0.05);

};

R_Date("VERA-5788", 16580, 70)

{

Outlier("General", 0.05);

};

};

Boundary("8");

Boundary("9");

Phase("Post Solutrean")

{

R_Date("VERA-5212", 12965, 40)

{

Outlier("General", 0.05);

};

};

Boundary("10");

};

Sequence()

{

Boundary("=3");

Date("La Boja Lower Solutrean");

Boundary("=4");

};

Sequence()

{

Boundary("=5");

Date("La Boja Upper Solutrean");

Boundary("=6");

};

Sequence()

{

Boundary("=7");

Date("La Boja Solutreo-Gravettian");

Boundary("=8");

};

Sequence("Total")

{

Boundary("=3");

Date("La Boja");

Boundary("=8");

};

};

**Parpalló**

Options()

{

Resolution=20;

};

Plot()

{

Outlier_Model("General",T(5),U(0,4),"t");

Sequence("PAP")

{

Boundary("1");

Sequence("Lower/Middle Solutrean")

{

R_Date("Birm-520", 20166, 380)

{

Outlier("General", 0.05);

};

R_Date("BM-859", 20490, 900)

{

Outlier("General", 0.05);

};

};

Boundary("2");

Boundary("3");

Sequence("Upper Solutrean")

{

R_Date("BM-861", 18080, 800)

{

Outlier("General", 0.05);

};

};

Boundary("4");

Boundary("5");

Phase("Solutreo-Gravettian")

{

R_Date("Birm-521", 17896, 340)

{

Outlier("General", 0.05);

};

R_Date("OxA-22651", 19020, 100)

{

Outlier("General", 0.05);

};

};

Boundary("6");

Boundary("7");

Phase("Magdalenian")

{

R_Date("Birm-519", 13960, 200)

{

Outlier("General", 0.05);

};

R_Date("OxA-22629", 18510, 100)

{

Outlier("General", 0.05);

};

};

Boundary("8");

};

Sequence()

{

Boundary("=1");

Date("Parpallo Lower/Middle Solutrean");

Boundary("=2");

};

Sequence()

{

Boundary("=3");

Date("Parpallo Upper Solutrean");

Boundary("=4");

};

Sequence()

{

Boundary("=5");

Date("Parpallo Solutreo-Gravettian");

Boundary("=6");

};

Sequence("Total")

{

Boundary("=1");

Date("Parpallo");

Boundary("=6");

};

};

**Mallaetes**

Options()

{

Resolution=20;

};

Plot()

{

Outlier_Model("General",T(5),U(0,4),"t");

Sequence()

{

Boundary("3");

Phase("Lower Solutrean / VI")

{

R_Date("MalladetesVI", 21710, 650)

{

Outlier("General", 0.05);

};

};

Boundary("4");

Boundary("5");

Phase("Middle Solutrean / Va")

{

R_Date("MalladetesVa", 20140, 460)

{

Outlier("General", 0.05);

};

};

Boundary("6");

Boundary("7");

Phase("Upper Solutrean / III")

{

R_Date("MalladetesIII", 16300, 1500)

{

Outlier("General", 0.05);

};

};

Boundary("8");

Boundary("9");

Phase("Solutreo-Gravettian / II")

{

R_Date("MalladetesII", 19370, 105)

{

Outlier("General", 0.05);

};

};

Boundary("10");

};

Sequence()

{

Boundary("=3");

Date("Mallaetes Lower Solutrean");

Boundary("=4");

};

Sequence()

{

Boundary("=5");

Date("Mallaetes Middle Solutrean");

Boundary("=6");

};

Sequence()

{

Boundary("=7");

Date("Mallaetes Upper Solutrean");

Boundary("=8");

};

Sequence()

{

Boundary("=9");

Date("Mallaetes Solutreo-Gravettian");

Boundary("=10");

};

Sequence("Total")

{

Boundary("=3");

Date("Mallaetes");

Boundary("=10");

};

};

**Cendres**

Options()

{

Resolution=20;

};

Plot()

{

Outlier_Model("General",T(5),U(0,4),"t");

Sequence()

{

Boundary("1");

Phase("Gravettian")

{

R_Date("Beta-155606", 24080, 150)

{

Outlier("General", 0.05);

};

R_Date("Beta-14283", 21230, 180)

{

Outlier("General", 0.05);

};

};

Boundary("2");

Boundary("3");

Phase("Upper Solutrean / XIII")

{

R_Date("Beta-118026", 18920, 180)

{

Outlier("General", 0.05);

};

R_Date("Beta-118027", 18750, 130)

{

Outlier("General", 0.05);

};

};

Boundary("4");

Boundary("5");

Phase("Solutreo-Gravettian / XII")

{

R_Date("Beta-118024", 17230, 130)

{

Outlier("General", 0.05);

};

};

Boundary("6");

};

Sequence()

{

Boundary("=3");

Date("Upper Solutrean");

Boundary("=4");

};

Sequence()

{

Boundary("=5");

Date("Solutreo-Gravettian");

Boundary("=6");

};

Sequence("Total")

{

Boundary("=5");

Date("Cendres");

Boundary("=6");

};

};
